# Neural Signature of Complex Daily Function in Early Dementia Risk: Brain Region and Spectral‐Specific Insights during Dual‐Task Walking

**DOI:** 10.1002/alz70856_102933

**Published:** 2025-12-25

**Authors:** Pierfilippo De Sanctis, Theo Vanneau, Wenzhu B Mowrey, Sophie Molholm, John J Foxe, Claudia Schneider, Jeannette R Mahoney, Jessica L Zwerling, Erica Weiss, Alexandria Hoang, Johanna Wagner

**Affiliations:** ^1^ Albert Einstein College of Medicine, Bronx, NY, USA; ^2^ University of Rochester Medical Center, Rochester, NY, USA; ^3^ Stony Brook University, Stony Brook, NY, USA; ^4^ Montefiore Einstein, Bronx, NY, USA; ^5^ Zander Labs, Delft, South Holland, Netherlands

## Abstract

**Background:**

Difficulties in performing complex everyday activities are a key component of diagnosing dementia syndromes. Subtle limitations in these functions have been observed even before a diagnosis of mild cognitive impairment. However, the neural correlates underlying functional decline, particularly during the early stages of dementia, remain poorly understood.

**Method:**

To address this gap, we utilized a dual‐task walking paradigm, portable electroencephalography (EEG), and 3D body tracking to record brain activity synchronized with cognitive and gait events in 36 individuals aged 65 and older. Participants were divided into lower‐risk (Montreal Cognitive Assessment [MoCA] ≥ 27, *n* = 18) and higher‐risk (MoCA ≤ 26, *n* = 18) groups for cognitive impairment (CI) using a median split. We assessed gait‐related brain activity in the 8–28 Hz range over the pre/postcentral gyrus, as a marker of sensorimotor activation, and in the 3–7 Hz range over the frontomedial cortex, as a marker of motor control. We hypothesized that higher CI risk would be associated with poorer performance and distinct fronto‐parietal activation patterns during dual‐task walking.

**Result:**

To address this gap, we utilized a dual‐task walking paradigm, portable electroencephalography (EEG), and 3D body tracking to record brain activity synchronized with cognitive and gait events in 36 individuals aged 65 and older. Participants were divided into lower‐risk (Montreal Cognitive Assessment [MoCA] ≥ 27, *n* = 18) and higher‐risk (MoCA ≤ 26, *n* = 18) groups for cognitive impairment (CI) using a median split. We assessed gait‐related brain activity in the 8–28 Hz range over the pre/postcentral gyrus, as a marker of sensorimotor activation, and in the 3–7 Hz range over the frontomedial cortex, as a marker of motor control. We hypothesized that higher CI risk would be associated with poorer performance and distinct fronto‐parietal activation patterns during dual‐task walking.

**Conclusion:**

By leveraging the high spatiotemporal resolution of EEG and 3D motion tracking to align brain activity with gait and cognitive events, this approach enables task‐specific, brain regional, and spectral insights into complex daily functions. This method holds significant potential to improve the prediction and identification of non‐invasive brain stimulation targets for interventions in early‐stage dementia syndromes.

